# Spin Waves in Ferromagnetic Nanorings with Interfacial Dzyaloshinskii–Moriya Interactions: II. Directional Effects

**DOI:** 10.3390/nano14030286

**Published:** 2024-01-30

**Authors:** Bushra Hussain, Michael Cottam

**Affiliations:** 1 Department of Natural Sciences, University of Michigan, Dearborn, MI 48128, USA; bhussai@umich.edu; 2 Department of Physics and Astronomy, University of Western Ontario, London, ON N6A 3K7, Canada

**Keywords:** spin waves, ferromagnetic nanorings, Dzyaloshinskii–Moriya interactions, dipole–dipole interactions, dipole-exchange modes

## Abstract

A theory is presented to study the effect of interfacial Dzyaloshinskii–Moriya interactions (DMIs) on the static and dynamic magnetic properties in single-layered ferromagnetic nanorings. A microscopic (Hamiltonian-based) approach is used that also includes the antisymmetric DMI besides the competing symmetric (bilinear) exchange interactions, magnetic dipole–dipole interactions, and an applied magnetic field. Here, the axial vector of the DMI is taken to be in the plane of the nanoring (by contrast with earlier studies) and we explore cases where it is either parallel or perpendicular to the in-plane magnetic field. Significantly, with this orientation for the DMI axial vector, the inhomogeneous static magnetization is tilted to have a component perpendicular to the plane giving a surface texture. This effect is studied in both the low-field vortex and high-field onion states. There is a consequent modification to the discrete set of spin-wave modes in both states through their frequencies and spatial amplitudes. We present combined analytical and numerical results for the static properties and dynamical magnetization in ferromagnetic nanorings, including the variation with applied field.

## 1. Introduction

The last decade has seen a tremendous activity in studying the antisymmetric exchange interactions, or Dzyaloshinskii–Moriya interactions (DMIs), in various magnetic nanostructures. While DMI phenomena were discovered long ago [1,2], there has been a recent upsurge in interest when it was found that the effects could be greatly enhanced by interfacing a ferromagnetic layer with certain heavy metal substrates (see, e.g., [3]). Typically, DMIs occur due to the presence of particular asymmetries in the crystal lattices of bulk magnetic materials (such as MnSi and FeCoSi) or when there is an interface between a ferromagnetic material and a heavy metal (e.g., the Mn/W or Fe/Ir systems). The antisymmetric exchange interaction can contribute to a distinctive behaviour in the static magnetization and the magnetic phases, and the spin-wave dynamics. For example, DMIs are known to produce chiral and topological features in nanostructures [4,5,6], opening up new perspectives for device applications. Also, regarding the spin dynamics in bulk-like materials with no interfaces taken into account, it has been pointed out and verified experimentally that contributions linear in the wave-vector components occur in the dispersion relations at small wave vectors [3,7,8,9,10,11,12,13], in addition to the quadratic contributions associated with the Heisenberg exchange. Several other studies have been conducted that elaborate on the wave-vector dependence of DMIs and their role in unidirectional spin-wave propagation and/or for Brillouin light scattering (BLS) (see [8,9,10,11,12,14,15,16]). The effects of DMIs on the statics and spin dynamics in ferromagnetic nanostructures have been reviewed, e.g., [3,4,17,18].

Although DMI studies have been directed towards several different magnetic nanostructures, such as stripes/wires, rings, disks, and the arrays thereof (see references above), the case of thin ferromagnetic nanorings deserves further attention due to the role of interfacial DMIs at the inner and outer radii. In the absence of DMIs, nanorings typically exhibit two states of stable magnetic ordering: the vortex state for a low field and the bidomain onion state for a high field [19,20]. This magnetic ordering is influenced by the interplay of the in-plane applied magnetic field with the exchange and dipole–dipole interactions. When the thickness of these rings is increased to become comparable with the radii, other magnetization states may occur [21]. Due to the potential for field-induced switching between the states, nanorings have become excellent candidates for switching and logic device applications [22,23]. It is important to have a thorough understanding of the magnetic states and spin-wave dynamics in nanorings for device applications such as spintronics, (bio) sensors, data storage, signal processing, and biomedical devices [22,23,24,25,26,27,28,29,30].

We recently carried out studies of the spin waves (SWs) in the vortex and onion states of nanorings using a microscopic, or Hamiltonian-based, method. Initially, the SW modes were investigated without DMIs [31]. Subsequently, a separate calculation was reported for nanorings with interfacial DMIs (or i-DMIs) included [32]. The latter paper (henceforth referred to as I), which is the forerunner to the present work, studied the case in which the directional properties of the interfacial DMIs were varied through making different choices for the axial vector. These directional effects were associated, in part, with the DMI axis being either in-plane or out-of-plane relative to the axis of circular symmetry for the nanoring. Our previous study (paper I) of DMIs in nanorings was focused on the case of the DMI axial vector perpendicular to the plane of the nanoring. The preferred orientations of the static magnetization remained in-plane, but they were modified in magnitude and direction, especially near the inner and outer radii due to the interfacial DMI effects. The transition field became shifted, while the frequencies and amplitudes of the SW states were sensitively modified.

By contrast, the focus in the present paper is the case in which the axial vector of the DMIs falls within the plane of the nanoring. As in paper I, we studied effects as the applied magnetic field was varied (including the switching between the vortex and onion states), so there were actually two in-plane situations that could now arise in general depending on whether the DMI axial vector was parallel to the in-plane applied field or perpendicular to that field. In related work, we note that Flores et al. [33] recently considered a vortex state in a large circular disk with an in-plane axial vector for the DMIs. In a macroscopic continuum model and in the absence of any applied magnetic field, they showed that the static magnetization vector was tilted out of the plane near the vortex core due to DMI effects.

In Section 2, we present the materials and methods employed in this study. Specifically, we describe the geometry and composition of the nanorings and the theoretical techniques employed. First, we examine the static behaviour of the inhomogeneous magnetization in a nanoring, allowing for the DMI axial vector in the plane of the nanoring to be either parallel or perpendicular to the applied magnetic field. We used a microscopic Hamiltonian approach that included the bilinear exchange, dipole–dipole interactions, Zeeman field energy, and the DMIs in a thin ferromagnetic nanoring geometry. Then, the theory for the magnetization dynamics in terms of spin waves is presented. The numerical results are given in Section 3, where it is shown that there was a novel tilting of the static magnetization, leading to a magnetic texture that was particularly evident near the inner and outer edges of the ring. As the applied field was increased through the phase transition into the onion state, the tilting effect became modified but was found to persist. Then, the theory for the magnetization dynamics is presented, incorporating the effects of the tilting. For the frequencies and spatial amplitudes of the SW modes, it was found that clear differences developed between the two cases for the in-plane DMI axial vector, both of which contrasted with the behaviour when the DMI axial vector was perpendicular to the plane (as in I). A discussion of the results and the overall conclusions are given in Section 4.

## 2. Materials and Methods

**Figure 1:**
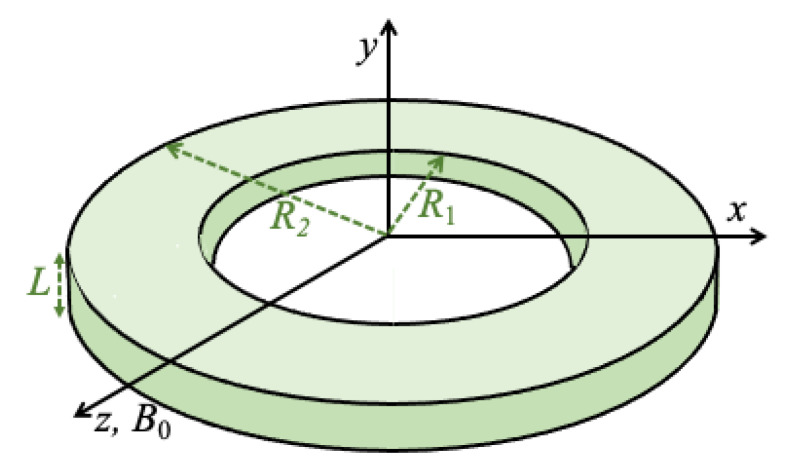
Geometry and choice of coordinate axes for the nanoring used in the DMI calculations. The inner and outer radii are $R_{1}$ and $R_{2}$, respectively, and the thickness is *L* (with $L\ll R_{2}$). The applied magnetic field $B_{0}$ is in the plane of the ring along the *z* direction and the DMI axial vector was chosen to be in the xz plane (see text).

The geometry of a circular nanoring in the xz plane is shown in Figure 1 where the inner and outer radii are $R_{1}$ and $R_{2}$, respectively, and the thickness in the *y* direction is L. Here, we assume flat rings with $L\ll R_{2}.$ The in-plane applied magnetic field $B_{0}$ is taken to be along the *z* direction. As in some of our recent work on the spin dynamics of nanorings and nanotubes (our preceding paper I and [31]), we employed a spin Hamiltonian together with a finite-element method. The spin sites form an array of elements filling the volume of the nanoring and were chosen to lie on a simple cubic lattice (with lattice constant *a*). In accordance with the established results from micromagnetic modelling, *a* must be chosen to be less than the so-called exchange correlation length $a_{ex}$, which is about 5 to 7 nm in metallic ferromagnets like permalloy or cobalt, for example).

We can express the total spin Hamiltonian as $H=H_{ring}+H_{DM}$, where $H_{ring}$ is the dipole-exchange part in the absence of DMIs [31] and $H_{DM}$ describes the additional DMI effects considered here. We write

(1) $$\begin{array}{ccc} H_{ring} & = & -\frac{1}{2}\sum_{n,m}J_{n,m}S_{n}\cdot S_{m}+\frac{1}{2}{\left(g\mu_{B}\right)}^{2}\sum_{n,m}\sum_{\alpha ,\beta }D_{n,m}^{\alpha \beta }S_{n}^{\alpha }S_{m}^{\beta }\\ & & -g\mu_{B}B_{0}\sum_{n}S_{n}^{z}, \end{array}$$

where $J_{n,m}$ is the symmetric Heisenberg-type exchange interaction between the spin sites labelled *n* and *m* with position vectors $r_{n}$ and $r_{m}$. We assume that it only couples with the nearest neighbours, with the value *J*, but otherwise, it is equal to zero. The long-range dipolar interaction coefficients $D_{n,m}^{\alpha \beta }$ between different sites have the form

(2) $$D_{n,m}^{\alpha \beta }=\frac{{\left|r_{n,m}\right|}^{2}\delta_{\alpha \beta }-3r_{n,m}^{\alpha }r_{n,m}^{\beta }}{{\left|r_{n,m}\right|}^{5}}$$

where α,β={x,y,z} and we define $r_{n,m}=r_{n}-r_{m}\neq 0$. The last term in Equation (1) represents the Zeeman energy of the applied magnetic field $B_{0}$ along *z*, with *g* and $\mu_{B}$ denoting the Landé factor and Bohr magneton.

The additional Hamiltonian $H_{DM}$ for the DMIs, with interfacial effects at the inner and outer radii of the nanoring, can be written in general as

(3) $$\begin{array}{ccc} H_{DM} & = & -\frac{1}{2}\sum_{n,m}J_{n,m}^{DM}d\cdot \left(S_{n}\times S_{m}\right), \end{array}$$

where d is the axial unit vector of the DMIs and $J_{n,m}^{DM}$ is the interaction strength (which satisfies the antisymmetry property $J_{n,m}^{DM}=-J_{m,n}^{DM}$ on interchange of the site labels). We assume nearest-neighbour exchange only, and we adopted the same sign convention as in [32] by labelling the value as $J^{DM}$ when the in-plane vector separation has a positive *x* or *z* component, and $-J^{DM}$ otherwise. An interior spin site in the nanoring will have four neighbours (with two neighbours of $J^{DM}$ and two of $-J^{DM}$), but spin sites near the lateral edges will have fewer neighbours, leaving the possibility of unmatched + and/or − pairs. There are consequences for both the static and dynamic properties of the nanorings, particularly at the edges, and these were investigated in [32] when the axial vector d is perpendicular to the plane of the nanoring. Here, we focus on the novel effects arising when d is in the plane, either along *x* (perpendicular to $B_{0}$) or along *z* (parallel to $B_{0}$). We note that, for a disk structure, Flores et al. [33] recently predicted that the presence of DMIs with an axial vector in the plane would cause the static magnetization to tilt out of the plane. Although the theoretical model here is different in several respects, including having a variable in-plane magnetic field $B_{0}$ to switch between vortex and onion states, we anticipated that tilting of the magnetization at the lateral edges in the nanoring might also occur.

The two cases under consideration are when the DMI axial vector is along *x* or *z*, giving

(4) $$\begin{array}{ccc} J_{n,m}^{DM}d\cdot \left(S_{n}\times S_{m}\right) & = & \left\{ \begin{array}{cc} J_{n,m}^{DM}\left(S_{n}^{y}S_{m}^{z}-S_{n}^{z}S_{m}^{y}\right), & \;d\|x\\ J_{n,m}^{DM}\left(S_{n}^{x}S_{m}^{y}-S_{n}^{y}S_{m}^{x}\right), & \;d\|z \end{array}.\right. \end{array}$$

We note in both cases that the right-hand side is linear in an $S^{y}$ operator, which suggests that the spins may become tilted (or canted) out of the xz plane for their equilibrium orientations at low temperatures $T\ll T_{C}$. To study this static behaviour, we follow a mean-field approach by writing $S_{n}=\left(S_{n}^{x},S_{n}^{y},S_{n}^{z}\right)=S\left(\sin\theta_{n}\cos\alpha_{n},\;\sin\alpha_{n}\sin\theta_{n},\;\cos\theta_{n}\right)$ for the spin vectors, where $\theta_{n}$ and $\alpha_{n}$ are spherical polar angles. Then, we may use the Hamiltonian to write down a total energy functional $\bar{E}$, which has the form

(5) $$\begin{array}{ccc} \bar{E} & = & -\frac{1}{2}\sum_{n,m}J_{n,m}\left(S_{n}^{x}S_{m}^{x}+S_{n}^{y}S_{m}^{y}+S_{n}^{z}S_{m}^{z}\right)-\frac{1}{2}\sum_{n,m}J_{n,m}^{DM}\left(S_{n}^{y}S_{m}^{z}-S_{n}^{z}S_{m}^{y}\right)\\ & & +\frac{1}{2}g^{2}\mu_{B}^{2}\sum_{n,m}(D_{n,m}^{x,x}S_{n}^{x}S_{m}^{x}+D_{n,m}^{x,z}S_{n}^{x}S_{m}^{z}+D_{n,m}^{y,y}S_{n}^{y}S_{m}^{y}+D_{n,m}^{z,x}S_{n}^{z}S_{m}^{x}\\ & & +D_{n,m}^{z,z}S_{n}^{z}S_{m}^{z})-g\mu_{B}B_{0z}\sum_{n}S_{n}^{z}, \end{array}$$

where we take the DMI axial vector along *x* and simplify it by setting terms $D_{n,m}^{xy}=D_{n,m}^{yz}=0$ by the symmetry for a nanoring with a thickness of just one cell in the *y* direction. A similar result can be written down for the case of the DMI axial vector along *z*. The components of the effective magnetic field can then be written down using $B_{n}^{\alpha }=-\left(1/g\mu_{B}\right)\;\delta \bar{E}/\delta S_{n}^{\alpha }$, where α={x,y,z}, yielding

(6) $$\begin{array}{ccc} B_{n}^{x} & = & \frac{1}{g\mu_{B}}\left[\sum_{m}J_{n,m}S_{m}^{x}-g^{2}\mu_{B}^{2}\sum_{m}\left(D_{n,m}^{x,x}S_{m}^{x}+D_{n,m}^{x,z}S_{m}^{z}\right)\right],\\ B_{n}^{y} & = & \frac{1}{g\mu_{B}}\left[\sum_{m}J_{n,m}S_{m}^{y}+\sum_{m}J_{n,m}^{DM}S_{m}^{z}-g^{2}\mu_{B}^{2}\sum_{m}D_{n,m}^{y,y}S_{m}^{y}\right],\\ B_{n}^{z} & = & B_{0}+\frac{1}{g\mu_{B}}[\sum_{m}J_{n,m}S_{m}^{z}-\sum_{m}J_{n,m}^{DM}S_{m}^{y}\\ & & -g^{2}\mu_{B}^{2}\sum_{m}\left(D_{n,m}^{z,z}S_{m}^{z}+D_{n,m}^{x,z}S_{m}^{x}\right)]. \end{array}$$

These coupled finite-difference equations can be solved numerically using iterative techniques, as described in earlier similar works (see, e.g., [31,32]). Briefly, this involves choosing a trial initial configuration for the set of angles. Next, the components of the effective magnetic fields are calculated using Equation (6), and each spin vector is reset to lie along the effective field direction just obtained. The process is then repeated iteratively with Equation (6) until convergence is obtained. In practice, several possible initial configurations are employed to avoid local energy minima. Then, the set of equilibrium angles $\left\{\theta_{n},\alpha_{n}\right\}$ can be deduced using relationships like $\cos\theta_{n}=B_{n}^{z}/\left|B_{n}\right|$ and $\tan\alpha_{n}=B_{n}^{y}/B_{n}^{x}$.

Having discussed the static magnetic properties, we now examine the magnetization dynamics in terms of the spin waves (SWs) in the nanorings. Following the approach in [31,32], for each spin at site *n*, we carry out a matrix transformation from the global (x,y,z) axes to a new set of local axes $\left(X_{n},Y_{n},Z_{n}\right)$ aligned with that spin such that the new $Z_{n}$ axis is along its equilibrium direction. Following [31,32], we rewrite the previous spin Hamiltonian in terms of boson operators using the Holstein–Primakoff transformation [34] applied relative to the *local* axes. Then, the Hamiltonian can be expressed in the form $H=H^{\left(0\right)}+H^{\left(1\right)}+H^{\left(2\right)}+\cdots$, where $H^{\left(i\right)}$ denotes a term with a product of *i* boson operators. The first term is a constant and does not influence the spin dynamics, whereas $H^{\left(1\right)}$ is zero by symmetry. The next term $H^{\left(2\right)}$ describes the linearized SWs and is our focus here, since we will ignore the higher-order terms that correspond to nonlinear processes.

The quadratic $H^{\left(2\right)}$ term has the form

(7) $$H^{\left(2\right)}=\sum_{n,m}\left\{A_{n,m}^{\left(2\right)}a_{n}^{†}a_{m}+B_{n,m}^{\left(2\right)}a_{n}^{†}a_{m}^{†}+B_{n,m}^{(2)*}a_{n}a_{m}\right\},$$

where $a_{n}^{†}$ and $a_{n}$ are the boson creation and annihilation operators at site *n*, respectively, and the coefficients of the operator terms can be separated into two parts as $A_{n,m}^{\left(2\right)}=A_{n,m}^{(2)ring}+A_{n,m}^{(2)DM}$ and $B_{n,m}^{\left(2\right)}=B_{n,m}^{(2)ring}+B_{n,m}^{(2)DM}$. Here, $A_{n,m}^{(2)ring}$ and $B_{n,m}^{(2)ring}$ refer to the contributions for a nanoring in the absence of the DMI effects; their explicit expressions are quoted in [31] in terms of the parameters of the Hamiltonian $H_{ring}$ in Equation (1). The terms $A_{n,m}^{(2)DM}$ and $B_{n,m}^{(2)DM}$ refer to the additional contributions from the DMIs with an in-plane axial vector; their contributions were found to be

(8) $$\begin{array}{ccc} A_{n,m}^{(2)DM} & = & \sum_{p}SJ_{n,p}^{DM}\left(\sin\alpha_{n}\sin\theta_{n}\cos\theta_{p}-\cos\theta_{n}\sin\alpha_{p}\sin\theta_{p}\right)\delta_{n,m}\\ & & +\frac{S}{2}J_{n,m}^{DM}\left(\sin\theta_{n}\cos\theta_{m}\cos\alpha_{m}-\cos\theta_{n}\cos\alpha_{n}\sin\theta_{m}\right),\\ B_{n,m}^{(2)DM} & = & \frac{S}{4}J_{n,m}^{DM}(\sin\alpha_{n}\cos\theta_{n}\sin\theta_{m}-\sin\theta_{n}\sin\alpha_{m}\cos\theta_{m}\\ & & -2i\sin\theta_{n}\cos\alpha_{m}), \end{array}$$

when the DMI axial vector is along *x*. Similarly, when the axial vector is along *z*, we found

(9) $$\begin{array}{ccc} A_{n,m}^{(2)DM} & = & \sum_{p}SJ_{n,p}^{DM}\sin\theta_{n}\sin\theta_{p}\left(\sin\alpha_{p}\cos\alpha_{n}-\cos\alpha_{p}\sin\alpha_{n}\right)\delta_{n,m}\\ & & +\frac{S}{2}J_{n,m}^{DM}\cos\theta_{n}\cos\theta_{m}\left(\sin\alpha_{m}\cos\alpha_{n}-\cos\alpha_{m}\sin\alpha_{n}\right),\\ B_{n,m}^{(2)DM} & = & -\frac{S}{4}J_{n,m}^{DM}\{\left(\cos\theta_{n}\cos\theta_{m}-1\right)\left(\cos\alpha_{n}\sin\alpha_{m}-\sin\alpha_{n}\cos\alpha_{m}\right)\\ & & +2i\cos\theta_{n}\left(\cos\alpha_{n}\cos\alpha_{m}+\sin\alpha_{n}\sin\alpha_{m}\right)\}. \end{array}$$

Following [31,32], the SW frequencies and amplitudes are found by diagonalizing $H^{\left(2\right)}$ using a generalized Bogoliubov transformation (as described in [35]). This leads to a dynamical block matrix defined by

(10) $$\left(\begin{array}{cc} A^{\left(2\right)} & 2B^{\left(2\right)}\\ -2B^{(2)*} & -{\tilde{A}}^{\left(2\right)} \end{array}\right),$$

where the tilde denotes a matrix transpose. The positive eigenvalues of the above large matrix correspond to the physical SW frequencies; there is a set of degenerate (in magnitude) frequencies formed by the negative eigenvalues. The “diagonalized” form of $H^{\left(2\right)}$ can be expressed as

(11) $$H^{\left(2\right)}=\sum_{l}\omega_{l}\;b_{l}^{†}b_{l}.$$

Here, the discrete spin-wave modes are denoted by $\omega_{l}$ where integer l=1,2,⋯ is a branch number, and $b_{l}^{†}$ and $b_{l}$ are the new boson operators for creation and annihilation of mode *l*. The eigenvectors of the matrix in Equation (10) yield the spatially dependent amplitudes [31,32] with the relative phase information included.

## 3. Results

The magnetic parameters required for the numerical illustration of the theory are the exchange stiffness D, the saturation magnetization $M_{s}$, and the gyromagnetic ratio $g\mu_{B}$. These parameters are related to those in the Hamiltonian by $D=SJa^{2}/g\mu_{B}$ and $M_{s}=g\mu_{B}S/a^{3}$. Also, D is related to the micromagnetic stiffness A by $D=2A/M_{s}$. For convenience, we took D = 30.0 T nm^2^, $M_{s}$ = 0.115 T, and $g\mu_{B}$ = 29.5 GHz T^−1^, which are values typical of permalloy or Ni_0.8_Fe_0.2_ (see, e.g., [36]), but our theory is of general applicability. Several values for the ratio $J^{DM}/J$ were taken to explore the range from −0.08 to 0.08. Different rings sizes were analyzed, where typically the outer radius $R_{2}=100$ nm and we employed different values for the wall width $t\equiv R_{2}-R_{1}$. The effective lattice *a* was chosen to be smaller than the exchange correlation length $a_{ex}$, which is ∼6 nm for the above parameters. Here, we chose a=4 nm. This ensures that the spin-wave frequencies obtained later are independent of the actual *a* value.

**Figure 2:**
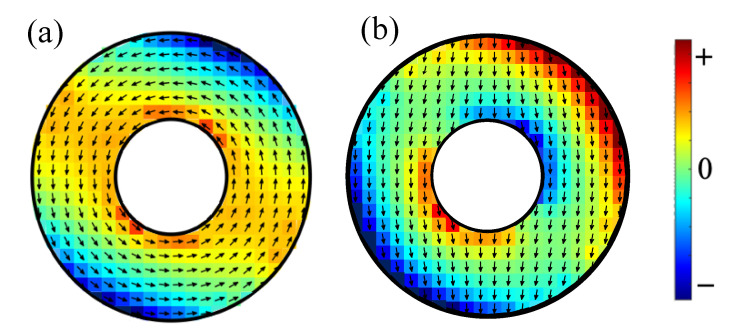
The equilibrium spin orientations (with in-plane component as shown by the arrows) for a small ferromagnetic nanoring with outer radius 40 nm, inner radius 16 nm, and thickness 4 nm in (**a**) a vortex state with applied field 0.005 T and (**b**) an onion state with applied field 0.05 T. The field direction (*z* axis) is downwards. The out-of-plane tilts up (+) and down (−) are represented by the colour coding. The DMI axial vector is along the *x* direction with $J^{DM}/J=-0.04$.

In order to illustrate the vortex and onion states as the field $B_{0}$ is varied, we first considered a smaller ring size, where $R_{2}=40$ nm, $R_{1}=16$ nm, and L=4 nm. Some results illustrating the equilibrium spin orientations for two field values are shown in Figure 2, depicting a flux-closure vortex state when $B_{0}=0.005$ T and a bidomain onion state when $B_{0}=0.05$ T. The in-plane components for the spin directions in each cell are represented by arrows, and colour coding is used to indicate the inhomogeneous tilting of spins as either up (+) or down (−) relative to the xz plane. It can be seen that the colour pattern is markedly different (with both radial and angular variations) for the two states and that the amount of tilt was generally greater near the lateral edges. These results were obtained for the DMI axial vector along the *x* direction.

**Figure 3:**
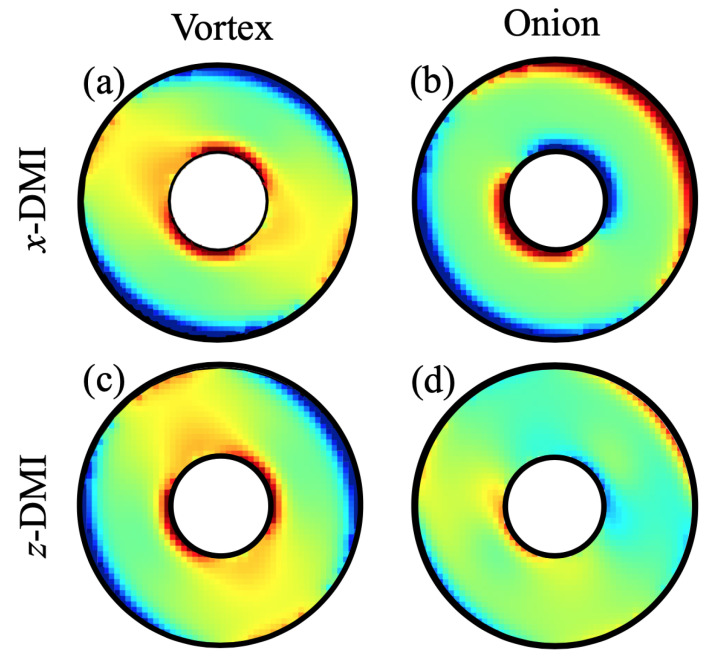
Colour plots showing the out-of-plane tilts as up (+) and down (−) for a nanoring with outer radius 100 nm, inner radius 36 nm, and thickness 4 nm. Panels (**a**,**b**) refer to the vortex (with $B_{0}=0.005$ T) and onion (with $B_{0}=0.03$ T) states, respectively, when the DMI axial vector is along the *x* direction. Panels (**c**,**d**) refer to the vortex (with $B_{0}=0.005$ T) and onion (with $B_{0}=0.03$ T) states, respectively, when the DMI axial vector is along the *z* direction. In all these cases, we chose $J^{DM}/J=-0.08$. The colour coding is the same as in Figure 2.

Similar colour plots are given next in Figure 3 for a larger ring size with $R_{2}=100$ nm, $R_{1}=36$ nm, and L=4 nm. Comparisons were made here between the tilt patterns in the vortex state (with $B_{0}=0.005$ T) and the onion state (with $B_{0}=0.03$ T), and between the DMI axial vector along the *x* and *z* directions. For the *x* case, as shown in Figure 3a,b, a comparison with Figure 2 indicates a broadly similar tilting pattern, but it is quite evident for the larger ring that the tilting was more localized near the lateral edges. A further comparison of panels (c) and (d) with panels (a) and (b) in Figure 3 shows how the colour plots were modified when the DMI axial vector was along the field direction *z*. The vortex cases (see Figure 3a,c)) are similar but with a rotation effect, whereas the onion cases (see Figure 3b,d) are significantly different with less tilting when the DMI axial vector was along the field direction *z*.

**Figure 4:**
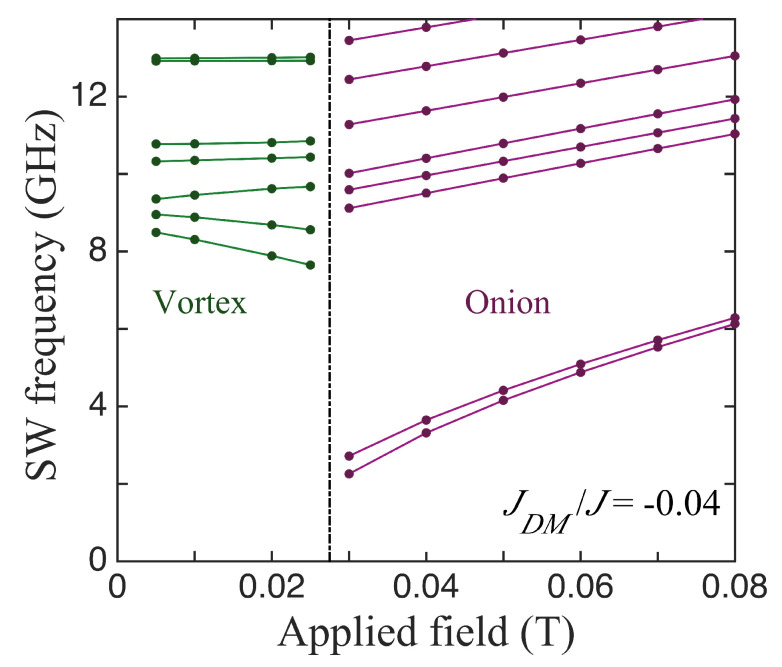
The SW frequencies plotted versus the applied field for the small nanoring (as in Figure 2) with outer radius 60 nm, inner radius 24 nm, and thickness 4 nm when the DMI axis is in-plane and along *x* (perpendicular to the field). The vertical line shows the transition field between the states. We took $J^{DM}/J=-0.04$.

We first present the results for the lowest few discrete SWs in the case of the smaller nanoring that was employed when studying the equilibrium orientations (see Figure 2). Now, in Figure 4, the calculated dependence of these SW frequencies on the applied field is shown. As expected, there was an abrupt change in the SW frequencies at the phase transition field (near 0.027 T) between the stable vortex and onion states. The main effects here, where the DMI had its axial vector along *x*, was that the mode frequencies, in general, were shifted due to the DMI and, specifically, there was an induced splitting in the lowest two SW branches in the onion phase. Next, we will describe this behaviour further for the larger ring sizes and include other choices for the DMI axial vector.

**Figure 5:**
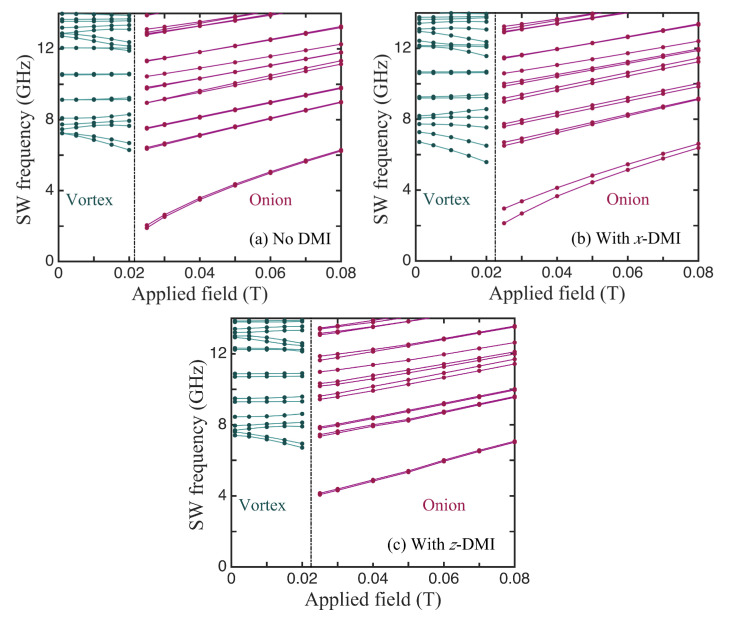
The SW frequencies plotted versus the applied field for a nanoring with outer radius 100 nm, inner radius 36 nm, and thickness 4 nm (as in Figure 3). Panel (**a**) shows the behaviour in the absence of DMIs ($J^{DM}/J=0$), while panels (**b**,**c**) correspond to nonzero DMIs ($J^{DM}/J=-0.08$) when the DMI axial vector is along *x* and *z*, respectively.

In Figure 5, we compare three cases for the SWs in ferromagnetic nanorings with an outer radius of 100 nm, an inner radius of 36 nm, and a thickness of 4 nm, for which the static behaviour was discussed earlier in Figure 3. The lowest SW frequencies plotted versus the applied field in the absence of DMIs are shown first in Figure 5a. The vertical line at $B_{0}≃0.022$ T indicates the transition field between the stable vortex and onion states. In the vortex case, we note that several of the discrete SW modes sloped downwards with the increasing field. This is typical of magnetic systems in which there are spins aligned both parallel and antiparallel to the field (e.g., antiferromagnets below the spin-flop transition) and is in accordance with previous theory [31] and Brillouin light scattering experiments [37] for nanorings. In the onion case, the SW frequencies all increased with the field. This is mainly due to the Zeeman term in the Hamiltonian, but it is noticeable that the lowest line in Figure 5a, which is actually two degenerate SW modes, has a convex curvature upwards because the magnetization is still reaching saturation. The modifications due to DMIs can be seen in Figure 5b,c, which correspond respectively to the in-plane axial vector being along *x* and *z*. All of the SW frequencies were shifted in general and there were seen to be splittings in some cases, where a degeneracy was removed. A clear example occurred in the *x*-DMI case where the doubly degenerate lowest frequency level in the onion state was split, with the effect being more pronounced just above the transition field (which now occurred when $B_{0}≃0.022$ T). By contrast, in the *z*-DMI case (where the DMI axial vector lies along the field direction), this degenerate lowest level underwent no significant splitting; in fact, its curvature reversed due to the DMI. Also, the transition field was effectively unchanged by the DMI. We have no obvious explanation for these differences, but they seem to be a consequence of the fact that, in the *x*-DMI case, the DMI energy in Equation (4) involves an $S^{z}$ spin component. This couples more strongly to the applied magnetic field via the Zeeman interaction.

**Figure 6:**
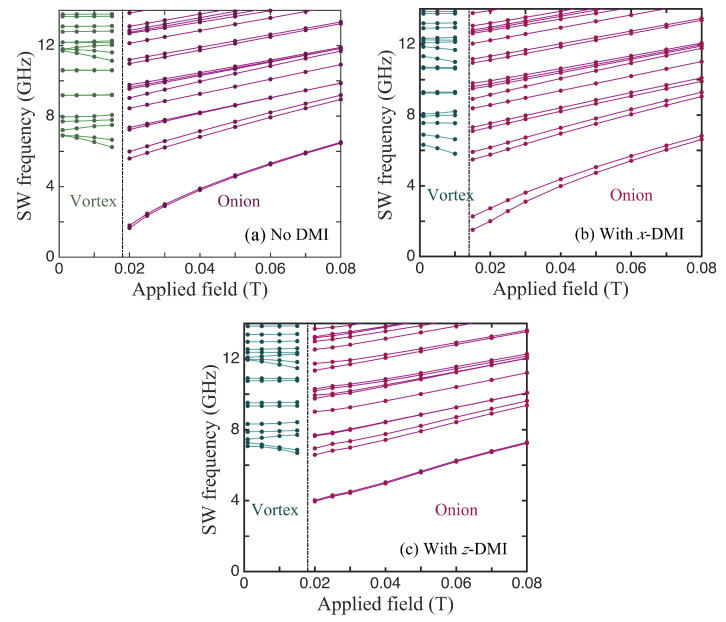
The same as in Figure 5 but for a nanowire with a larger wall width of 76 nm (with outer radius 100 nm, inner radius 24 nm, and thickness 4 nm).

Then, in Figure 6, we show some further results for a nanoring with larger value of the wall width $t=R_{2}-R_{1}$. Specifically, we take a ferromagnetic nanoring with an outer radius of 100 nm, an inner radius of 36 nm, and a thickness of 4 nm, and we again compare the SW frequencies in the absence of DMIs (see Figure 6a), with *x*-DMI present (see Figure 6b), and with *z*-DMI present (see Figure 6c). Although the frequencies are shifted compared with Figure 5 and the transition fields are lower in value, the effects due to DMIs were found to be qualitatively very similar for the two ring sizes.

**Figure 7:**
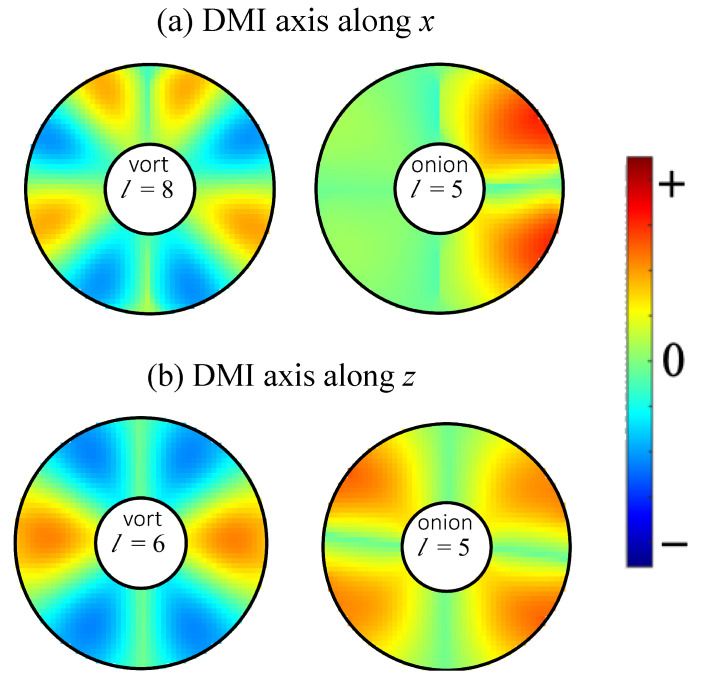
Colour plots of SW amplitudes for a nanoring with outer radius 100 nm, inner radius 36 nm, and thickness 4 nm (as in Figure 3 and Figure 5) for the DMI axial vector (**a**) along *x* and (**b**) along *z*. Examples for a vortex state (with $B_{0}=0.005$ T) and an onion state (with $B_{0}=0.03$ T) are shown. The mode numbers *l* are as labelled. The corresponding frequencies of the modes are 10.61 and 7.79 GHz from left to right in (**a**) and 9.30 and 7.97 GHz from left to right in (**b**).

Finally, we present some results for the SW amplitudes, which, as explained earlier, can be obtained from the eigenvectors of the dynamical matrix defined in Equation (10). For any eigenvalue (SW frequency), the different elements of the corresponding eigenvector refer to the relative amplitude factors in the different cells of the effective spins. These give us the spatial variations displayed as the colour plots in Figure 7. There are examples for SW modes in both the vortex and onion states and for both *x*-DMI and *z*-DMI. The same nanoring as in Figure 5 (with $R_{1}=36$ nm, $R_{2}=100$ nm, and L=4 nm) was assumed. Relative phase effects are included in these plots, so a difference in sign means a phase difference of 180°. In all cases, we observed a strong azimuthal (angular) dependence, especially as the mode number *l* increased. In the radial direction, the amplitudes tended to be largest in magnitude near the outer lateral edge. Some asymmetric amplitude distributions occurred for pairs of modes that were close in frequency. The mode in Figure 7a for l=5 and f=7.79 GHz in an onion state provides an example for this; its counterpart (not shown here), which is left–right reversed, occurred for l=6 and f=7.95 GHz. The lowest frequency modes corresponding to l=1 or 2 (amplitude plots not shown here) were similar to the “uniform mode” of SW precession and showed much weaker amplitude variations than those illustrated.

## 4. Discussion

In this paper, we present a study of the effects of *in-plane* interfacial DMIs (i-DMIs) on the static (equilibrium) properties and the magnetization dynamics in unsaturated ferromagnetic nanorings, contrasting with the results in our previous paper (paper I) [32] for the case where the DMI axial vector was perpendicular to the plane of the nanoring. As before, we employed a microscopic (or Hamiltonian-based) approach to obtain a dynamic matrix, given by Equation (10) in the present case, whose eigenvalues and eigenvectors provide the required results for the frequencies and the spatial distribution of amplitudes (including the phase) of the SW modes. By changing the in-plane applied magnetic field, we explored the magnetic behaviour in the rings from the low-field vortex state to the high-field onion state.

A major point of novelty in the present work concerns the directional effects associated with the different cases that may arise for the DMI axial vector. Here, we studied the in-plane (xz-plane) orientations for the axial vector (either along or perpendicular to the applied field). Both cases were found to show that out-of-plane tilting occurs for the equilibrium (static) spin orientations. This was expected for any general ferromagnetic material exhibiting i-DMIs, since we used only the exchange stiffness, saturation magnetization, and gyromagnetic in the numerical examples given. This effect due to the interfacial DMI was particularly evident at the edges near the inner and outer radii of the rings, as shown in Figure 2 and Figure 3. The tilting did not occur in the case of the preceding paper (paper I) where the DMI axis was in the out-of-plane direction (along the *y* direction). The tilting of the equilibrium spin directions had a consequent effect on the SW excitations, and this property was incorporated into our formalism in Section 2 and then into the numerical results presented in Section 3. We note that the two cases considered here of the DMI axis lying along *z* or along *x* are different because of the in-plane magnetic field (along *z*) that drives the transition between the vortex and onion states.

In this study, we illustrate how the novel tilting effects due to the in-plane DMI were localized near the inner and outer radii of a nanoring and display interesting angular variations. Generally, as the inner radius $R_{1}$ was reduced to a small value, we found that the localization became more pronounced. A practical lower limit occurred, however, when $R_{1}∼2a$ because difficulties arose in how to treat the vortex core. It is hoped that this theoretical study of the directional effects, which has identified interesting behaviours, will prompt some experimental investigations. While it is unlikely that the direction of the interfacial DMI can be practically controlled, the extensive literature cited in Section 1 provides numerous examples of the occurrence of i-DMIs. Depending on the combination of the materials (and presumably on the symmetries and other properties associated with the interface), different directional effects may be encountered. The case of an in-plane axial vector for the DMI was envisioned, for example, by Moon et al. [3], in the context of a different geometry from the nanoring system considered here. Typically, they considered a trilayer with a nonmagnetic top layer providing spin-orbit coupling, a ferromagnetic layer, and then a different symmetry-breaking nonmagnetic substrate layer. Specifically, an example was given [3] for an infinitely long chain of spins oriented parallel to the interfaces. In our case, the enhancement of the symmetry-breaking effect (and hence the DMI coefficient) might be achieved by applying an in-plane strain to the substrate in the nanoring system.

Several developments of the present work would be of interest for future investigations. One extension would be to apply our SW results to experimental data, such as those derived from BLS and microfocused BLS measurements (if these data become available) for nanorings that display well-characterized DMIs. Further, our DMI calculations could be extended to apply to thick rings (or equivalently to finite-length nanotubes), or to nanorings where an out-of-plane magnetic field has been applied (e.g., as in recent experimental studies [38,39] conducted in the absence of DMIs). Another possibility would be to study magnonic crystals formed by arrays of nanorings.

## Data Availability

All of the data present in this paper will be made available upon reasonable request. Please contact the corresponding author for further information.
